# The Physiology of the Circulation

**Published:** 1860-01

**Authors:** John C. Dalton

**Affiliations:** Professor of Physiology and Microscopic Anatomy


### NEW YORK MONTHLY REVIEW

AND

### BUFFALO MEDICAL JOURNAL.

**VOL. 15.** JANUARY, 1860. **NO. 8.**

### ORIGINAL COMMUNICATIONS.

ART. I.—*The Physiology of the Circulation. A Course of Lectures delivered at the College of Physicians and Surgeons, New York, in the Fall Term of* 1859. By JOHN C. DALTON, JR., M. D., Prof. of Physiology and Microscopic Anatomy.

### LECTURE III.

(SEPTEMBER 23.)

Movements of the Heart during Life.—Appearance of the Lungs in Respiration.—Alteration of Blood in passing through the Lungs.—Alternate Action of Auricles and Ventricles.—Elongation, hardening and twisting of the Heart.—Effects of stopping Respiration.—Change of color in Heart and Blood.—Distention of Right Auricle.—Of Right Ventricle.—Of Left Ventricle and Auricle.—Passage of Venous Blood into Left Cavities.—Distention of Aorta and Arterial System.—Order and Mechanism of these Changes.—Distribution of Venous Blood throughout Arterial System.—Condition of Heart and Vessels at time of Death.—Post-mortem Changes.—Final stoppage of Circulation.

HERE, gentlemen, is a full-grown dog, in whom, as you see, the chest has been opened, and the action of the lungs and heart exposed to view. A short time ago the animal was poisoned by the inoculation of woorara, and the nozzle of a bellows being immediately afterward inserted into the trachea, artificial respiration has been kept up; so that the circulation is now maintained in nearly its natural condition, and with its natural vigor.

We can therefore examine, at our leisure, the action of the heart and the play of the lungs. We shall afterward study the influence of various respiratory conditions upon the cardiac movements.

The lungs, you observe, in the first place, present a different hue according to their condition of collapse or distention. When filled with air, they are of a very light, pale rosy color—almost white; but when allowed to collapse, they become darker in hue, owing to the larger proportion of blood which they contain at that time. The color of the lungs, however, is at all times much lighter and paler than would usually be supposed by one who had only seen them as they appear after death.

In the natural condition of the parts, the anterior edges of the lungs overlap considerably the heart, so as to cover most of its surface; but by gently drawing them aside we can uncover very fully the heart and the large vessels in connection with it. In this animal, the pericardium has also been cut away, and the adipose tissue about the bases of the pulmonary artery and aorta carefully dissected off, so that the arterial trunks are fully exposed at their origin and easily distinguished from each other.

As the edges of the lungs are now drawn away in this manner, notice, if you please, the situation of the different parts of the heart and the course of the blood through them. Here, upon the right, you have the ascending and descending venæ cavæ through which the blood passes in two rapid and somewhat wavy streams, which meet in the cavity of the right auricle. The right auricle shows very distinctly the thinness of its walls, and in many places, particularly in the appendix auricularis, the bundles of muscular fibres are so far separated from each other that the transparent endocardium and the dark color of the venous blood may be easily distinguished between them. Below and in front of this portion, you see the right ventricle, with the conus arteriosus passing upward and to the left, and conveying the blood through the pulmonary artery to the lungs. The ascending portion of the arch of the aorta emerges from behind the base of the pulmonary artery, but soon becomes the more prominent vessel of the two, in the median line, to nearly the upper extremity of the sternum.

On turning over the heart toward the right side, the left auricle and ventricle, with the termination of the pulmonary veins, come into view. In the natural position of the organ, it is only the edges of its left chambers which can be seen from the front, their bodies being mostly concealed behind the right cavities and large vessels.

Now direct your attention, in the next place, gentlemen, to the effect of respiration on the color of the blood, as it passes through the heart and the pulmonary circulation. Here is the right auricle. It contains, as you know, the blood which has just arrived from the general system, by the great veins; and the color of this blood, distinctly seen through the auricular walls, is a dark bluish purple. On the opposite side of the heart, in a corresponding situation, is the left auricle, containing the blood which has just returned from the lungs. The color of this blood, also seen through the walls of the auricle, is of a brilliant scarlet, strongly contrasting with that on the opposite side.

We see here, then, in the living body, the change of color which characterizes the blood of the arterial and venous systems; and we are enabled to distinguish the exact point in the circulatory system at which this change takes place. The blood, immediately before its entrance into the lungs, is blue. Immediately after leaving them, it is red; and this change is incessantly repeated, as fresh portions of blood arrive at the right auricle and ventricle, pass through the pulmonary circulation, and return to the left cavities of the heart.

You observe, however, that while there is this difference in hue between the right and left auricle, there is no such difference in the color of the two ventricles. The ventricles both present a clear, fresh red tinge, which is of precisely the same shade on the left side as on the right.

This is because the color presented to the eye by the auricles and the ventricles depends upon different conditions. In the auricles, the thinness and transparency of their walls, as we have already seen, allow the color of the blood which they contain to show through them; and the right auricle, accordingly, appears blue, while the left is red. In the ventricles, on the other hand, the muscular parieties are so thick and opaque, that they conceal entirely the color of the blood in their cavities, and show only the tinge of that which is circulating in their capillary vessels, and is intermingled with their muscular fibres. Both ventricles have the substance of their walls supplied by the same arterial blood, and consequently present the same general tinge externally. The color of the auricles, accordingly, is due to the color of the blood contained in their cavities; while that of the ventricles is due to the color of the blood circulating in their capillary vessels.

Such are the general appearances and color of the lungs and heart, in the natural condition of the circulatory apparatus.

Let me now call your attention, gentlemen, to several of the most important phenomena relating to the action of the heart and its different portions.

And first, as to the alternate movement of the auricles and ventricles.

The action of the entire organ consists, as you observe, in a series of constantly recurring and alternating contractions and relaxations. But it is easy to see, in the outset, that there is no very marked distinction in time between the contraction of the auricle and that of the ventricle. At least, there is no perceptible interval between these two occurrences. Although you see that the auricular contraction comes first, you nevertheless cannot perceive any actual pause or separation between it and the ventricular systole. The contraction of the heart has, therefore, the character of a continuous and peristaltic movement, which commences at the base of the organ with the contraction of the auricles, and runs immediately forward from them to the apex of the ventricles.

It is almost unnecessary to point out that, while this is going on, the two auricles always act simultaneously with each other, and the two ventricles also perform their contraction at the same time upon the right and left sides.

The next point to observe particularly is the hardening of the heart at the time when the ventricles contract. This hardening can be very readily felt by the finger, laid upon the surface of the organ, or by the hand grasping its substance. The fibres of the heart, like those of any other muscle, grow rigid during contraction, and the whole organ assumes a condition of firmness and tension. The hardening of the heart, therefore, is simultaneous with the contraction of its ventricles and the discharge of their contents. At the same time, the organ performs a well-marked spiral or rotatory movement, upon itself, directed from left to right. The spiral fibres and their contraction show themselves most distinctly just at the apex of the organ. At that point they are seen converging to a centre, and producing a kind of dimple, at each contraction of the heart, by their twisting or rotatory motion.

These three kinds of simultaneous action—the peristaltic contraction of the ventricles, the rigidity or tension of the heart, and its twisting motion from left to right—are the first phenomena relating to the cardiac movements which we have now to observe.

The next is that the heart, at the moment of the ventricular contraction, *elongates*. The action of the spiral and circular fibres of the ventricles is to draw the sides of the organ together at its base, and to shoot out or protrude the apex. As the apex starts forward in this way, it strikes against the walls of the chest, and produces the impulse or "shock" of the heart, so called, which is felt externally. It is easy to see here that the finger, laid upon the point of the heart, is lifted with violence at every contraction of the ventricles. It is still more readily observed, when the heart is lifted gently so that the apex points upward, and then viewed from the direction of the diaphragm. The posterior surface of the organ, which is thus brought into view, has a very regular triangular shape, and shows very distinctly, at each pulsation, a narrowing of the base and elongation of the point.

Such are the principal points with regard to the action of the heart in a natural condition of the circulation and respiration, to which I shall ask your attention to-day. Let us now pass to the consideration of the phenomena presented by the circulatory apparatus when the respiratory movements are disturbed or suspended.

You observe, that while the circulation is going on in a natural manner, and while the lungs are alternately filled and emptied by the movements of respiration, the color of the blood, in passing through these organs, is completely changed from blue to scarlet. The blood is arterialized while traversing the pulmonary circulation. It is converted from venous blood, which you see here in the right auricle, to arterial blood, which you see in the left auricle; and the substance of the heart itself is of a clear rosy tinge, owing to the bright arterial blood circulating in its capillaries.

I will now stop the artificial respiration, which has been kept up thus far, and allow the lungs to collapse. A change already begins to take place in the color of the heart. Its tinge is less brilliant and more dusky than before; and the longer respiration is allowed to remain suspended, the deeper and more bluish it becomes. The surface of the heart is now of a very dark, uniform purplish hue, which pervades the whole substance of the organ, and gives it an appearance quite different from that which it presents in a normal condition. The left auricle, too, which before presented a clear scarlet tinge, is now perfectly blue, and there is no difference in the color of the blood which shows through its walls and that contained in the cavity of the right auricle.

I now recommence the movements of respiration, and almost instantly you see a bright flush of the natural ruddy color, spreading over the surface of the heart, and the entire organ is rapidly restored to its normal appearance. The left auricle, also, has regained its original rosy hue, and the whole circulation goes on as before.

This change of color in the heart, in consequence of a stoppage of respiration, is due to a change of color in the blood. This fluid being no longer arterialized in its passage through the lungs, blue venous blood is infiltrated throughout the substance of the heart, and communicates to it its own purplish hue. This is the first and most palpable effect which is produced by a stoppage of the respiration.

But there are other effects due to the same cause, to which I will now direct your attention. These are the distention of the cavities of the heart and accumulation of blood in different parts of the circulatory system, in consequence of a disturbance of the respiratory movements.

I now suspend once more the artificial respiration, and you will see the change in shape and size of the organ, produced by distention of its cavities.

At first, little or no alteration is to be observed in this respect; but after the lapse of a minute or two, you already see that the right auricle begins to be enlarged. This enlargement continues to increase as the color of the heart grows darker, and at last becomes very great, and the organ begins to labor under the load of accumulated blood. The auricle is now excessively distended, its sides bulging out far beyond their natural limits; and the heart is fast approaching that condition in which a continued accumulation would paralyze its muscular fibres, and finally arrest its pulsations.

The distention of the right auricle, in consequence of a stoppage of the respiration, is a circumstance which has been long known to physiologists; but what I wish you to notice particularly here is the fact, that this distention is not confined to the right auricle, but *extends also to the right ventricle.* The ventricle, as you see, is now, in point of fact, distended in the same way as the auricle. The whole right side of the heart is loaded with venous blood, and suffers in the same manner from its accumulation. When the movements of respiration are allowed to recommence, and the blood comes back arterialized from the capillaries of the lungs, the ventricle again begins to discharge its contents;—it has already relieved itself of a portion of the load, and gradually returns to its original size.

Now, this is an effect which I wish to notice particularly, in the first place, because it is a part of the mechanism of the heart's action, under these circumstances, which is not fully understood, and which is not always described with perfect accuracy. When respiration is suspended, the venous blood accumulates on the right side of the heart, in both cavities; and the right ventricle becomes distended almost simultaneously with the right auricle. In point of fact, in viewing the actual experiment, we find that the distention of the ventricle takes place, if anything, a little earlier than that of the auricle. At any rate, it is certainly not perceptibly later.

This fact is the more important to establish here, because it is one, I think, not generally appreciated. Almost invariably, where you see the effect of a stoppage of respiration on the heart's action discussed, it is the distention of the right auricle which is insisted on, but not that of the right ventricle. In the case of Mr. Groux, for example, to which I alluded in the last lecture (fissure of the sternum), the fact that the pulsating tumor in the medio-sternal space became enlarged during a prolonged expiration was regarded by some observers as a proof that it was formed by the auricle and not by the ventricle. But from what we have just seen it is evident that the distention of the ventricle, in such a case, may be equally prominent with that of the auricle, and, in fact, would be more likely to cause a protrusion in the median line.

But we have thus far ascertained only a part of the phenomena which occur when the action of the heart is disturbed from stoppage of the respiration.

If we now repeat again the experiment of suspending the respiratory movements, you will see that it is not only the right auricle and ventricle which become enlarged from an accumulation of the blood, but *the left auricle and ventricle become distended also.* The cavities on both sides of the heart are gorged with blood to an equal, or nearly equal degree. If, while the right cavities are now beginning to enlarge, you watch the left edge of the heart, you will observe that an enlargement is taking place as well in the left cavities;—and if we now compare the two sides together, we should say, strange as it may seem, that the left ventricle is even more distended than the right. This fact introduces a new element into our explanation of the heart's action, and requires us to examine the theory of its mechanism a little more closely.

At first—so long as it was supposed that the distention of the heart, under stoppage of respiration, was confined to its right cavities—this distention was thought to be accomplished by a very simple process. It was explained in this manner. After the stoppage of respiration, the function of the lungs ceasing, the circulation in the pulmonary capillaries was also supposed to come to an end at the same time;—and the blood, stagnating in the vessels of the lungs, thus exerted a backward pressure upon the right cavities of the heart, which, in consequence, became distended and overloaded. This backward pressure was communicated, it was thought, to the large veins near the heart, and through them to the entire venous system—so that the blood accumulated in the veins and the right side of the heart, simply because it could not pass through the pulmonary capillaries, and thus reach the arterial system.

Now, this is really the case, to a certain degree, as we have already seen. At least, such an accumulation actually takes place upon the right side of the heart. But it takes place in the ventricle as well as in the auricle; and the distention of the ventricle is, if anything, prior to that of the auricle, showing that it is produced by a backward pressure. It is a kind of regurgitation from the lungs, owing to an obstacle in the pulmonary circulation.

Such is the idea most commonly entertained with regard to the production of these phenomena.

But this explanation will not account for all the appearances which are observed. For you have seen that the distention of the heart shows itself equally upon both sides; or, if anything, the left auricle and ventricle are distended more than the right cavities. How are we to account for this distention of the left cavities? Is it that the blood is expelled in large quantities from the lungs, and so accumulates in the left cavities of the heart, not being able to pass through them? This can not be the correct explanation; for we have seen that the blood, so far from passing in greater abundance through the lungs after respiration is suspended, passes less readily than usual. There is an absolute obstacle to its flow through the pulmonary vessels. It can not, therefore, be the blood coming from the lungs which distends the left cavities of the heart, so much as an accumulation of blood which retrogrades, or which tends to retrograde, from the general circulatory system backward to the left cavities.

Now, we may convince ourselves that this is in reality the case, by examining the order in which the changes just mentioned present themselves. To illustrate this, I will repeat some experiments which I have recently performed in reference to this subject. From their results you will see reason to believe that the distention of the left side of the heart, after stoppage of the respiration, is a distention which takes place from before backward—that is, a distention from the general system toward the left ventricle and auricle. One of these experiments was performed in April last, in the presence and with the assistance of Dr. Sands, of New York, and Dr. Da Costa, of Philadelphia.

*Experiment.*—April 5th, 1859. A full-grown dog was poisoned with woorara. The chest was then opened, and artificial respiration kept up by a bellows inserted into the trachea.

The following observations were then made on the changes in the appearance and action of different parts of the heart, after suspending the artificial respiration—the lungs being allowed to remain, during each trial, in a state of complete collapse:

I. The rapidity of the heart's action, immediately after the stoppage of respiration, is the same as before, viz., twenty-eight beats in ten seconds.

II. The right auricle begins to swell distinctly before the left auricle.

III. Both ventricles swell moderately soon after the stoppage of respiration, but we cannot tell which, if either, begins to enlarge first.

IV. On the right side, the ventricle and auricle both become distended—we think, but without being too positive, the ventricle first.

V. On the left side, we are sure that the ventricle begins to swell before the auricle.

VI. The pulmonary artery begins to swell a considerable time before the pulmonary veins or the left auricle. The pulmonary veins and the left auricle swell at a later period, nearly at the same time, but the auricle a little the first.

VII. Blue blood appears in the pulmonary veins and the left side of the heart, before there is any *excessive* swelling of any part of the organ.

You observe here, gentlemen, that the order of distention of the cardiac cavities is always from before backward. On the right side the auricle and the ventricle become distended nearly at the same time—but the ventricle, if anything, rather the first, and on the left side the ventricle and auricle enlarge before the veins; while the pulmonary artery enlarges considerably before the pulmonary veins and the left auricle. Everywhere, accordingly, the ventricle swells before the auricle, and the auricle before the veins; and we shall soon see that the great arteries leading from the heart swell before either.

This, then, leads us, in the course of our examinations, to a new and very remarkable fact, which has hitherto entirely escaped observation. It is this—that not only do the right ventricle and pulmonary artery become distended almost at the same time with the right auricle, and not only do the left cavities of the heart become distended in the same, or nearly the same degree with the right cavities—but the *aorta itself*, and the commencement of the arterial system, become distended from a similar cause, at the same time.

This fact of the distention of the aorta is of great importance, as it furnishes the key to the whole mechanism of the influence of the stoppage of respiration.

You will find that the order in which these changes present themselves, after the lungs are allowed to collapse, is as follows. There are two distinct stages in this process, marked by essentially different phenomena. In the first period there is a *very slight* swelling of the cavities of the heart, which takes place almost immediately after the stoppage of respiration. At this time, blue blood begins to pass through the capillaries of the lungs, and to appear in the left side of the heart. This is a point which I wish you to notice particularly. After respiration has stopped, the circulation through the lungs does not stop—but, on the contrary, the blood continues to pass through the capillaries of these organs. The only difference is that under these circumstances the blood is no longer arterialized. But, so far from the pulmonary circulation being arrested by this, the blue blood passing through the lungs, and still retaining its venous character, begins to pass through the pulmonary veins into the left cavities of the heart. I have found, in experiments directed to this point, that after the suspension of respiration, the unaërated blood passes through the lungs so rapidly as to fill the cavity of the left auricle in thirty seconds. The venous blood thus passes into the left auricle, from the left auricle to the left ventricle, and from the left ventricle to the aorta, whence it is distributed through the arterial system.

Now comes the second stage of the process. As soon as the venous blood has fairly found its way into the arterial system, there begins an excessive distention of the cavities of the heart, *but preceded by a distention of the arterial system and the aorta.* The arterial system and the aorta become engorged because the venous blood which they contain, arriving at the capillaries, is not fit to circulate through them. It accordingly stagnates at the capillary system, and immediately begins to exert a backward pressure upon the arteries. From the arterial system the engorgement extends to the aorta, and from the aorta to the left cavities of the heart. The blue blood which continues to pass through the lungs, and of which the arterial system cannot disgorge itself, accumulates consequently in the aorta and in the left cardiac cavities.

We will now repeat, in this animal, the experiment of stopping the respiration, as the heart, I presume, has now completely recovered from the effects of the last trial. The two auricles, right and left, contain now, as you see, respectively blue and scarlet blood. The two ventricles present their natural size and action, and the pulmonary artery and aorta are seen above, the aorta occupying precisely the median line, the pulmonary artery running obliquely upward and a little to the left. The aorta, as you also see, is very slightly distended at each pulsation of the ventricles, and reacts by its own elasticity, immediately afterward. On touching it with the end of the finger it is felt to be tolerably firm, but elastic and flexible, and it can be partially compressed by the finger without much difficulty.

Now, on stopping the movements of respiration, we see reproduced all the appearances which we have heretofore noticed, and in addition this new phenomenon of the arterial distention. The blood soon passes in an unaërated condition into the left cavities of the heart. The left auricle already contains so much dark blood that you now see little or no difference in color between it and the right auricle. The heart begins to be distended also at this time, upon the left side as well as upon the right; and both the left cavities, auricle above and ventricle below, are now distended, not with arterial, but with venous blood.

But while these changes are going on, the aorta also, you observe, suffers an alteration in size. This swelling of the aorta is very manifest before there is any excessive enlargement of either the right auricle on the one side or the pulmonary veins on the other. When the heart begins to labor under the accumulated blood, the variation in size of the distended aorta at each pulsation becomes much more marked. At each contraction of the ventricles it is now excessively distended, and then partially collapses, though it constantly remains unnaturally enlarged. If the finger be laid upon it, it is felt to be excessively tense and resisting, and to have lost the flexibility and compressibility which belong to it in the natural condition.

A recommencement of the respiratory movements again restores the normal state of the circulation, and the heart and pulmonary artery and aorta gradually relieve themselves of blood.

It is evident, therefore, that the distention of the aorta precedes that of the other central organs of circulation. The following experiment will illustrate some of the more essential particulars in reference to this point.

*Experiment.*—Sept. 9th, 1859. A dog was inoculated with woorara three and a half hours after feeding. The chest was opened, and artificial respiration kept up through the trachea.

The lungs were then allowed to collapse, and the effect upon the circulatory organs noted as follows:

I. Unaërated, blue blood passes through the lungs, so that the blood in the left auricle is completely dark-colored in thirty seconds.

II. There is a *slight swelling* of both right and left cardiac cavities soon after the suspension of respiration, as observed in previous experiments.

III. Some minutes afterward, the heart begins to labor, and a marked and excessive *distention* of the same cavities takes place, both auricles and both ventricles being filled with dark blood.

IV. But the pulmonary artery and the aorta are also distended at this second stage of the process, and their distention is equally marked with that of the ventricles. The ascending portion of the aorta becomes distended to $\frac{1}{4}$ more than its original diameter; and the pulmonary artery to nearly $\frac{1}{8}$ more than its original diameter. The distention of the aorta is both more marked and more promptly produced than that of the pulmonary artery, and even *precedes, if anything, the excessive distention of the ventricles.*

V. At the time also that the aorta is so enlarged, there is *no sensible distention of the venæ cavæ*, in the neighborhood of the heart.

VI. After this state of things has continued for a short period the aorta becomes more excessively distended at the time of each cardiac contraction, and again partly collapses at the time of relaxation; the blood evidently regurgitating then from the aorta into the ventricle.

VII. After artificial respiration is recommenced, bright scarlet blood passes round into the left auricle, so as completely to restore its natural color in fifteen seconds. The aorta and pulmonary artery remain distended and indurated for a few minutes, but gradually return to their original size and softness.

VIII. If respiration be allowed to cease with the lungs kept moderately full of air (by tying the trachea after an insufflation), all the above phenomena follow, as described, but at later periods. For example, blue blood begins to fill the left auricle, in this instance, only after about two minutes. The other changes also are proportionately delayed, but are not essentially different in character from the foregoing.

Now, there are one or two points in these experiments which I wish to notice more particularly than we have yet done. They relate to the manner in which the circulation of the blood, or its continuous passage through the vascular system, becomes disturbed and finally arrested. For there is a period, immediately after stoppage of the respiration, during which the chemical changes of aëration and arterialization of the blood are suspended, but in which its continuous movement still goes on as before. The auricle drives the blood into the ventricle, and the ventricle into the aorta; and when the aorta is expanded by the impulse of the cardiac contraction, its subsequent reaction drives the blood which it has received onward toward the capillaries. Each successive portion of the heart and arteries impels its volume of blood farther and farther upon its course, and thus contributes to keep up the incessant round of the circulation. Even the blue, unaërated blood, returning from the lungs, passes from the auricle into the ventricle, from the ventricle into the aorta, and from the aorta into the arterial system.

But soon afterward the distention of the aorta begins to show itself, which we have already noticed; and, at the same time that it begins to fill with venous blood, its pulsations become more marked and vibratory. They are more distinctly visible to the eye, and more perceptible to the touch. Now, why is this? It is for the same reason that the pulsations of an artery became more marked after it is tied, as in surgical operations, amputations, and the like. There is an obstacle to the onward passage of the blood which did not exist before; and the influence of this obstacle is felt in an increased tension and vibration of the artery.

After a short period, however, the distention of the aorta becomes excessive, and then a change is at once perceptible in the efficiency of the heart's action. Its contractions become labored, and, as it were, spasmodic, with longer intervals between them. At each contraction of the ventricle there is an increased expansion of the aorta with the new volume of blood driven into it. But when the aorta reacts after this expansion, the blood is no longer driven forward as before, and distributed through the arterial system, but regurgitates through the aortic orifice and again fills the relaxed ventricle. It is then evident that the heart fails to maintain the circulatory movement of the blood. The obstacle to its passage in the arterial system is too great to be overcome by the cardiac impulse, and the natural continuous flow of the blood through the vascular system is replaced by an alternating flux and reflux, from the ventricle into the aorta, and from the aorta back again into the ventricle.

Another exceedingly remarkable fact in this connection, is that the great veins, as we have seen, in the neighborhood of the heart, do not participate in this engorgement of the ventricle and the aorta. All the cavities of the heart may be enlarged and full of venous blood. The aorta also may be enlarged, and crammed to its utmost tension with the same venous blood, while at the same time the venæ cavæ are hardly, if any, larger than in the natural condition of the circulation.

We are now able, I think, to understand what takes place in the circulatory system, when the aëration of the blood is interfered with. In point of fact, after the stoppage of respiration, the venous blood, which naturally belongs only to the venous system, passes round into the arterial system. It can pass through the capillaries of the lungs, it can find its way into the left cavities of the heart, and from the left cavities of the heart it can find its way into the arterial system, but from the arteries it *cannot* find its way through the capillaries of the general system. Here is the obstacle which opposes the passage of the impure blood, and which arrests the continuous movement of the circulation.

Accordingly, we find that in such an experiment it is not the great veins which become distended after stoppage of the respiration. The disturbance which results is not principally because the blood finds a difficulty in passing through the lungs, and so distends the right auricle and the great veins, and finally presses backward upon the capillary system. This is the manner in which the process is usually described; but you see it is not the way in which it actually takes place. The order and direction of these changes, on the contrary, is precisely the opposite. The reaction takes place, not from the great veins to the capillaries, and thus through the capillaries to the arterial system; it is the arterial system which first becomes distended, then the aorta, then the left cavities of the heart. While the whole arterial system, accordingly, is filled and distended with venous blood, which the capillaries will not allow to pass, and while the whole heart, auricles and ventricles, is distended in the same manner, the venæ cavæ, both superior and inferior, as we have seen, are not particularly enlarged, and in fact are rather less abundantly supplied with blood than usual.

I will now demonstrate, by again stopping the respiratory movements in this animal, that under these circumstances, the blood not only passes through the lungs to the left cavities of the heart, but is also distributed from the left cavities throughout the entire arterial system.

I make an incision here over the situation of the right femoral artery, the chest remaining open, and the thoracic organs exposed, as before. I now open the sheath of the vessel, taking care not to wound the small muscular branches which you see passing off toward the inner and outer part of the thigh. Here you have the femoral artery fully exposed. Now, on stopping the respiration the pulsations of the vessel become very soon much more strongly marked than before. The artery raises itself visibly at each pulsation and exhibits a tremulous movement, owing to the greater obstacle to the flow of blood which is established by the want of aëration. It is already, also, assuming a darker color, showing that the blood which circulates in it is acquiring a venous tinge instead of the bright arterial hue which belongs to it. Blue blood in fact, has at this moment passed into the left cavities of the heart, and the aorta is beginning to be distended and indurated. I now open the femoral artery by a transverse section, and you see the blood which flows from it is perfectly venous in character.

On recommencing the movements of respiration, the tension of the artery is very soon diminished, and in a few seconds, as you see, it again contains arterial blood, with a perfectly natural ruddy tinge.

In that short space of time, therefore, the venous blood had penetrated throughout the arterial system, and was afterward rapidly replaced by arterial blood, as soon as the function of respiration was re-established.

The following are the phenomena, accordingly, which show themselves in the circulatory organs, after stoppage of the respiration. First, passage of unaltered venous blood through the lungs, to the left side of the heart. Second, entrance of this venous blood into the aorta, and its general distribution over the arterial system. Arrived at the capillaries, the venous blood first meets with a serious obstacle to its passage. What is the precise nature of this obstacle, we do not as yet understand; but it is certain that the venous blood does not pass freely and readily through the capillaries of the general system, but is blocked and stagnated in this part of its course, and that then by a backward pressure it begins to engorge and distend the arteries. We cannot suppose, however, that the venous blood is absolutely and entirely arrested in the capillaries, but only that its passage through them is rendered much more slow and difficult than before. For when respiration again begins, as we have seen, and the blood is aërated at the lungs, though the entire arterial system be at that moment full of venous blood, arterial blood will yet in a few moments find its way into the arteries everywhere from the heart outward, and restore the natural course of the circulation. The venous blood, in this case, must escape through the capillaries in order to give place to the aërated and arterialized fluid.

The obstacle to the passage of venous blood through the capillaries, therefore, is partial, not complete. But it is still sufficient to produce an immediate backward engorgement of the arterial system. Then the aorta becomes distended at its origin, and the left ventricle and left auricle in succession, being unable to relieve themselves of blood through the arterial system, become distended in a similar manner. During this time, the same kind of engorgement takes place in the pulmonary artery and the right cavities of the heart;—though the distention of the pulmonary artery is never so excessive as that of the aorta, either because there is less obstacle to the passage of venous blood through the lungs than through the general capillaries, or because the injecting force of the right ventricle is less than that of the left, or because less blood is supplied by the capillaries to the veins, and by the veins to the right side of the heart. In either case, the principal accumulation is certainly in the arterial system.

Soon afterward the heart begins to labor in its pulsations, and the blood regurgitates, between the pulsations, from the aorta into the ventricle. The excessive accumulation then paralyzes the contractile power of the cardiac walls, and death takes place with all the cavities of the heart distended with venous blood.

Let us now see what further changes take place at the moment of death, when the circulation actually comes to an end. For though the appearances just described are those which follow directly upon a stoppage of the respiration, they are not such as present themselves in a postmortem examination, some hours later. An alteration must, therefore, take place in the course of the phenomena, at some time either during or after the death of the animal. Observation shows that this alteration begins to take place when the heart ceases to act.

The periods which we have thus far examined, have been characterized by an accumulation of venous blood in the arterial system and left cardiac cavities, followed by an imperfect action of the heart and regurgitation of blood from the aorta into the ventricle. The arterial system is at this time distended to its utmost. For the venous blood, which it already contains, can only with the greatest difficulty pass through the capillaries, and the powerful contraction of the left ventricle drives into it at each impulse a fresh supply. This state of things continues, so long as the action of the ventricle continues sufficiently vigorous to give the blood an impulse toward and into the arteries. But the continued distention, which the heart suffers, soon begins to paralyze its action more effectually; and at this time it can be seen that *the engorgement of the arterial system diminishes, while that of the heart increases.* The regular, rythmical action of the left ventricle stops. It no longer discharges any blood into the aorta; and the aorta and arteries generally, by the elastic resiliency of their own walls, and the slow passage of the venous blood through the capillaries, gradually relieve themselves of the load which they contained, and return to their original size. They even become smaller than in the natural condition of the circulation, for the blood of which they relieve themselves is now no longer replaced by fresh supplies from the lungs and heart. The fibres of the heart during this time exhibit an irregular, tremulous, vermicular contraction, which is to be seen first in the auricles, and afterward in the ventricles, but which is entirely insufficient to produce any proper contraction of the cardiac cavities, or any impression on the blood which they contain. The left auricle is at this period enlarged so as to be quite equal in size to the right; and of the two auricles, both of which are then of precisely the same color, the left is very much more tense and firm than the right. The pulmonary veins are also quite tense, while the venæ cavæ, on the right side of the heart, are even then hardly enlarged beyond their natural size. The left auricle, however, in the last stages of the heart's action, diminishes sensibly in size, while the right auricle remains distended; and the venæ cavæ in the immediate neighborhood of the heart fill up more or less with the blood which has come round to them from the capillary system. The pulmonary artery remains distended longer than the aorta, but finally relieves itself, and loses altogether its unnatural size and tension.

At the moment of death, accordingly, when all action of the heart has finally ceased, the arteries are empty or nearly empty of blood, and the venæ cavæ in the cavity of the chest are moderately engorged. The right auricle and right ventricle are distended with blood, and the left auricle is reduced to about half the size of the right; while the left ventricle is still also distended, rounded and bulging in shape, elastic and fluctuating to the touch, and, like the right ventricle, full of liquid venous blood. This condition of the heart can be seen at any time by allowing the respiration and circulation to come to an end in the manner which I have just described.

But after death another change takes place, which produces a still further alteration in the relation of the parts. This consists in a post-mortem contraction of the walls of the heart, showing itself most prominently in the left ventricle, which is the most muscular and powerful part of the organ. This alteration in the condition of the heart is undoubtedly similar in its nature to the cadaveric rigidity which shows itself, some time after death, in the external muscles of the trunk and extremities. It is the cadaveric rigidity of the heart. But it may be established much more promptly than the corresponding condition of the external muscles. I have seen the left ventricle of a dog, one hour after death, quite firm and resisting to the touch, when no sign of cadaveric rigidity could yet be perceived in the limbs; and even three and four hours after death there may yet be no distinct cadaveric rigidity in the external muscles, while this muscular induration is very fully developed in the left ventricle.

The effect of this postmortem contraction of the cardiac fibres is to obliterate the cavity of the left ventricle and to empty it of blood. A similar effect is produced, to a certain extent, in the right ventricle, which perceptibly diminishes in size within a few hours after death. But the muscular walls of this portion of the heart are too thin, and their action too feeble, to overcome the accumulation, and the right ventricle remains permanently distended with a considerable quantity of blood. The left ventricle, however, continues to contract, until it is very much reduced in size, and its sides brought nearly or quite in contact with each other.

This fact explains why it is that although the left ventricle is full of blood at the moment of death, it is almost invariably found to be empty in a postmortem examination made some hours subsequently. Its postmortem contraction gradually forces the blood from its cavity into the arterial system, and the arteries, retaining their elasticity, continue to force it onward through the capillaries, as before. This passage of the blood, therefore, which is accomplished during life by a series of successive, vigorous, cardiac pulsations, takes place after death by a slow and imperceptible postmortem contraction of the ventricle, analogous to the cadaveric stiffening of the voluntary muscles. The blood which it contained filters slowly through the arterial and capillary systems, and appears finally in the venous trunks and branches. Here it accumulates, by gravitation, in the most dependent parts of the body, and in those veins which are not compressed by the muscles in a state of rigidity.

The great veins of the chest and abdomen thus become the principal receptacles for the blood after death. The venæ cavæ themselves, in the immediate neighborhood of the heart, have even seemed to possess in a slight degree the property of postmortem contraction, and to exhibit accordingly, like the right auricle and ventricle, traces of cadaveric rigidity. The presence of striped muscular fibres in the walls of the veins in that situation, is sufficient to explain the existence of such a property. Notwithstanding this, the venæ cavæ in the cavity of the chest still remain quite full of blood, which accumulates, however, to the most remarkable degree in the portal vein, and in the abdominal vena cava below the liver.

If the thoracic organs accordingly be examined some hours after death, when cadaveric rigidity is fully established, the left ventricle and auricle are found to be very much contracted and indurated, and nearly empty, containing not more than one-quarter or one-eighth part as much blood as the corresponding cavities on the opposite side. The aorta is also very much diminished in size, and nearly empty; while the venæ cavæ, and particularly the inferior vena cava in the abdomen, are distended much beyond their natural size. There is a certain amount of induration in the right auricle and right ventricle; but these cavities are still full of blood, and comparatively soft and yielding to the touch. The pulmonary artery contains only a moderate quantity of blood, though more in proportion than the aorta; while the pulmonary veins are full. The blood, of course, under these circumstances, is everywhere perfectly dark and venous in color.

We thus see how it is that, some time after death, the blood is collected mostly in the right side of the heart and the large veins, although the immediate effect of a stoppage of respiration is to produce an engorgement of the arterial system and the left cardiac cavities.

I will now, gentlemen, stop altogether the artificial respiration in the animal which we have been using for experiment; and we can then notice in their order all the phenomena which present themselves, and see how the action of the heart finally comes to an end. The left cavities already contain dark blood, which passes through into the arterial system. Now the heart begins to labor, and at the moment this takes place, the distention of the aorta commences to show itself.

In the second stage of the process, which is now beginning, you see the increased and labored action of the cardiac cavities, the aorta expanding widely at each pulsation, and then partly emptying itself, by regurgitation, into the ventricle. You notice also, at this moment, that notwithstanding the immense distention of the cardiac cavities, the venæ cavæ are not particularly enlarged. It is the arterial system which is engorged at this period, not the venous.

The contractions of the heart are very slow, but they are still perfectly regular, and have otherwise their natural character, with the exception that they are more forced, and recur with less rapidity than usual. If respiration were to be re-established in this condition of the circulatory organs, I presume we might still recall the heart to its normal activity.

But if you allow the suspension of respiration to continue beyond a certain limit, even for a few seconds, you will find the heart paralyzed; and that paralysis having once taken place, the action of the organ cannot be recalled, although the lungs be brought again into play.

While the pulsations of the heart are now going on very slowly, you will observe a very curious fact, which was not perceptible when the organ acted with its normal rapidity, viz: a distinct interval of time between the contractions of the auricle and ventricle. The whole order of the heart's action, in fact, can be more easily analyzed, when it is thus retarded by an obstruction to the circulation. The auricle can be seen to contract first, and then the contraction of the ventricle follows after a slight interval.

The heart has now arrived at that condition that the circulation of the blood is no longer accomplished. Though the ventricles still contract, yet their contractions are not sufficient to discharge the blood which they contain, and force it into the arteries. Accordingly, the distention of the arterial system, you see, has already very much diminished, and the aorta has nearly returned to its original size. How does this happen? How has the artery got rid of its contents? Partly, it is evident, by a regurgitation of blood into the ventricle, which no longer fills up the artery by its own contraction, and partly by a slow filtering away of the blood through the capillary system into the veins.

The contraction of the cardiac fibres is now excessively irregular, and operates only in such a way as to distend the various parts of the organ by alternate and altogether local contractions and relaxations. These movements are no longer harmonious with each other, and of course are entirely inefficient to produce any definite effect on the circulation.

These irregular and localized movements of the cardiac walls continue for some time, becoming more feeble and uncertain, until they finally come to an end. The contraction of the ventricles ceases before that of the auricles, and that of the left auricle before that of the right; in which, as you are aware, the natural irritability lingers for a longer period than anywhere else. Finally, the activity of every part of the organ is exhausted, and all indications of vitality come to an end.

